# Gibberellin-induced parthenocarpy in fruits of a prickly pear mutant

**DOI:** 10.1007/s00299-025-03568-w

**Published:** 2025-08-06

**Authors:** Rameshkumar Ramakrishnan, Udi Zurgil, Shamili Kanna, Danuše Tarkowská, Ondřej Novák, Miroslav Strnad, Noemi Tel-Zur, Yaron Sitrit

**Affiliations:** 1 French Associates Institute for Agriculture and Biotechnology of Drylands, The Jacob Blaustein Institutes for Desert Research, Ben-Gurion University of the Negev, Sede Boqer Campus, 84990 Beer-Sheva, Israel; 2 Laboratory of Growth Regulators, Faculty of Sciences, Palacký University & Institute of Experimental Botany AS CR, Šlechtitelů 27, 783 71 Olomouc, Czech Republic; 3 Katif Research Center for R&D, Ministry of Innovation, Science and Technology, 8771002 Netivot, Israel

**Keywords:** *Opuntia ficus-indica*, Prickly pear, Gibberellins, Ovule, Cacti, Parthenocarpy

## Abstract

**Key message:**

A parthenocarpic fruit mutant of prickly pear was isolated, revealing the role of GAs in parthenocarpic fruit development which is controlled by the GID-GA20ox/GA2ox genetic system modulating GA biosynthesis/regulation.

**Abstract:**

We explored the intricate dynamics of parthenocarpic fruit development in prickly pear *Opuntia ficus-indica* (Cactaceae) through the investigation of fruits of the Beer Sheva1 (BS1) a parthenocarpic mutant and its revertant non-parthenocarpic stems. BS1 fruits, characterized by parthenocarpy and enlarged unfertilized ovules, provide a unique model for investigating the regulatory mechanisms underlying fruit development in prickly pear. We hypothesized that elevated levels of gibberellins (GAs) in BS1 ovaries induce parthenocarpic fruit development. By integrating different approaches, including GA quantification and expression analysis of ovaries from BS1 and revertant flowers, we elucidated the pivotal role of biosynthetic, catabolic, and regulatory GA genes in orchestrating ovule development. Notably, our investigation revealed a complex interplay between GA biosynthesis and catabolic genes, particularly GID1, GA20ox, and GA2ox, which significantly influenced GA levels in BS1 ovaries. Quantification of endogenous GAs confirmed higher levels of bioactive GA1, GA3, and GA4 in BS1 compared to revertant ovules, indicating the central role of GAs in parthenocarpy. Furthermore, application of the GA inhibitor paclobutrazol (PBZ) to BS1 flower buds resulted in the reversion of BS1 fruits to the progenitor phenotype containing viable seeds, thereby validating the critical involvement of GAs in seed development. High-throughput RNA-sequencing analysis identified a total of 7717 differentially expressed genes (DEGs) in BS1, among them GA-related genes. Overall, our findings shed light on the complex hormonal regulatory network governing parthenocarpic fruit development in prickly pear, paving the way for future studies aiming at understanding ovule development and development of commercially desirable seedless fruits.

**Supplementary Information:**

The online version contains supplementary material available at 10.1007/s00299-025-03568-w.

## Introduction

Global warming and its consequences have promoted increased interest in developing new crops that are highly adapted to harsh climate conditions. One such economically valuable crop species is prickly pear, *Opuntia ficus-indica* (Cactaceae), whose crassulacean acid metabolism (CAM) photosynthetic mechanism confers the advantage of improved water usage efficiency, which enhances the plants’ ability to survive droughts. The fruits of this species have a delicious juicy pulp, but their acceptability for human consumption is limited due to the presence of many hard-coated seeds (10–15% of the fruit pulp). Hence, acquiring a comprehensive knowledge of seed development in prickly pear—as a means of reducing the seed content of the fruit—is imperative for future promotion of this promising crop.

In plant reproduction, the central processes of fruit and seed development are usually determined by pollination and fertilization (Mesejo et al. 2016; Jiang et al. 2020). In some cases, however, plants can produce seedless parthenocarpic fruits without ovule fertilization (Li et al. 2014; Galimba et al. 2019) for example, in some varieties of cucumber, banana, apple, tomato, and citrus (Li et al. 2014; Mignolli et al. 2015; Mesejo et al. 2016; Galimba et al. 2019). In parthenocarpic fruits, imbalances in the levels of hormones—such as cytokinins, auxins, gibberellins (GAs), and brassinosteroids—compensate for the lack of seed-produced hormones, thereby enabling fruit set (Fos et al. 2000; Serrani et al. 2007; Mesejo et al. 2016; Liu et al. 2018; Sharif et al. 2022). Parthenocarpy can also be induced artificially by exogenous application of such hormones or genetic manipulations (Neji et al. 2017; Sharif et al. 2022). Whether natural or induced, parthenocarpy is both an essential instrument for investigating seed and fruit development (Fos et al. 2000; Galimba et al. 2019) and a desired trait in many crops.

With the above considerations in mind, we investigated a spontaneous mutant of prickly pear, known as Beer Sheva1 (BS1), that produces parthenocarpic fruits. This mutant was discovered among plants of the commercial cultivar ‘Ofer’ growing in Beer-Sheva, Israel (Weiss et al. 1993). The mutant plants produce flowers with enlarged ovules that develop into degenerated seed-like structures—some small, and others huge and stony. Notably, some new stems (~ 10% of the total stems) of the BS1 plant bear normal revertant flowers (the ‘Ofer’ progenitor phenotype) containing small ovules, and these plants set fruit with normal viable seeds (Weiss et al. 1993). Other distinctive characteristics of BS1—vs. revertant—fruits are earlier fruit ripening, a longer fruit neck, and fewer and smaller spines on the peel (Weiss et al. 1993). In addition, at the time of anthesis, BS1 ovules are five times larger than those in revertant fruits or in ‘Ofer’ fruits (Weiss et al. 1993). The distinctive fruit phenotype of BS1 implies that elevated levels of GAs may exist in BS1, which enable the development of parthenocarpic fruit. This premise is supported by previous studies on prickly pear showing that application of GA_3_ reduced the number of seeds (Marini et al. 2020) and promoted parthenocarpic fruit development (Neji et al. 2017). Similarly, in a different cactus species, *Opuntia amyclaea*, GA_3_ treatment also induced the development of parthenocarpic fruit (Mejía and Cantwell 2003). Further support may be drawn from our amplified fragment length polymorphism (AFLP) analysis comparing BS1 and revertant DNAs from the stems of the same plant (truly isogenic lines), which revealed sequence homology for transposable elements and GA-related GRAS transcription factors, thereby indicating that transposable elements could be involved in changes of GA metabolism/signaling in BS1 plants (Sitrit, unpublished data).

The key components of GA signaling are the gibberellin-insensitive DWARF1 **(**GID1) receptor, DELLA repressors, and F-box proteins (Zentella et al. 2007; Liu et al. 2018; Shinozaki et al. 2020). GID1 plays a dual role in the GA signaling pathway, functioning as both a GA receptor and an activator of genes involved in GA synthesis, such as gibberellin 20-oxidase (GA20ox) and gibberellin 3-oxidase (GA3ox) (Hedden 2020). GID1 binds to GA to form a complex that binds strongly to DELLA proteins. The GID1-GA-DELLA complex is recognized by F-box proteins, which promote the degradation of DELLA proteins, leading to the activation of genes involved in GA synthesis (Ueguchi-Tanaka et al. 2005). The removal of DELLA proteins triggers several GA-responsive processes, including the transition to flowering, pollen tube growth, and cell expansion during fruit development (Fuentes et al. 2012; Shinozaki et al. 2020). Importantly, the reduction in DELLA activity promotes parthenocarpic fruit development (Martí et al. 2007; Fuentes et al. 2012). Among the many relevant examples of DELLA activity cited in the literature, we mention just a few: the silencing of DELLA genes in tomato plants induced facultative parthenocarpic fruits and produced slender plants with long flower trusses (Martí et al. 2007; Jiang et al. 2020). In seedlings of *Arabidopsis thaliana*, microarray studies identified the GA-positive regulator *SCARECROW*-*LIKE* (*SCL*)* 3* as a gene that directly targets DELLA, thereby attenuating DELLA activity (Zentella et al. 2007). SCL3, which shares similarities with the GA-regulator Scarecrow (SCR) (Zhang et al. 2011), acts as an antagonist to DELLA in maintaining GA homeostasis through a feedback loop that regulates the expression of GA biosynthetic genes. In summary, the level of biologically active GAs in tissues is controlled by the expression of genes responsible for GA synthesis and degradation, and the expression of those genes is manipulated by the GA signaling pathway (Hedden 2020; Wang et al. 2020), as elaborated below.

GA biosynthesis occurs via the terpenoid pathway. In the first three steps of this process: (i) GGPP (trans-geranylgeranyl diphosphate) is converted to *ent*-kaurene in several steps that take place in plastids; (ii) then, *ent-*kaurenic acid is converted to GA_12_—again in several steps—in the endoplasmic reticulum; and (iii) finally, diverse GA derivatives are produced from the resulting GA_12_ in the cytosol via two branches of the pathway (Sakamoto et al. 2004; Shinozaki et al. 2020; Hedden 2020), the non-13-hydroxylation and the early 13-hydroxylation branches, as follows: the bioactive hormones GA_4_ and GA_34_ are formed from GA_12_ in the non-13-hydroxylation branch, and the bioactive GA_1_ is formed from GA_12_ via the enzymes GA13oxs and GA20oxs in the non-13-hydroxylation pathway and the early-13-hydroxylation branches, respectively. In both branches, GA3oxs produces the bioactive forms, GA_4_, GA_1_, and GA_3_. In addition, GA_9_ and GA_20_ can be converted to the bioactive forms GA_7_ and GA_3_, respectively (Hedden 2020). In the GA biosynthesis pathway, seven essential enzymes are necessary to produce GAs; they can be divided into two groups: early-step enzymes, namely, copalyl diphosphate synthase (CPS), *ent*-kaurene synthase (KS), kaurene oxidase (KO), and *ent*-kaurenoic acid oxidase (KAO), and later-step enzymes, GA20ox, GA3ox, and GA2ox (Sakamoto et al. 2004; Wang et al. 2020). Compared to early GA biosynthetic genes (coding for CPS, KS synthase, and KO), the later-stage genes (coding for GA20ox, GA3ox, and GA2ox) have a direct impact on the levels of bioactive GAs and on the plant phenotype in many plant species (Fleet et al. 2003; Chai et al. 2019). The later-step genes thus play pivotal roles in regulating the content of active GAs (Fleet et al. 2003; Shinozaki et al. 2020), while GA deactivation is controlled by different GA2ox enzymes in both branches, leading to the production of GA_8_ and GA_34_ catabolites (Rieu et al. 2008; Dorcey et al. 2009).

The genes involved in the GA biosynthetic and catabolic pathways play an important role in the development of parthenocarpic fruit, whether occurring naturally or induced by hormone manipulation (Wang et al. 2020). There are many examples in the literature describing the three main methodologies for altering the GA balance in the flowers of fruits species. The first approach relies on gene manipulation to induce parthenocarpy. For example, in parthenocarpic tomato *Solanum lycopersicum* L., GA biosynthetic genes, such as *GA20ox1*, *GA20ox2*, and *GA3ox1*, were upregulated (García-Hurtado et al. 2012). In the Chinese white pear *Pyrus bretschneideri*, parthenocarpic fruit development was induced via overexpression of *GA20ox2*, which enhanced GA_4_ synthesis (Wang et al. 2020). In fig *Ficus carica*, parthenocarpic fruit development was induced by upregulating *GA20ox*, *GA3ox*, *GID1* and *GID2* and downregulating the GA-inactivation gene *GA2ox* (Chai et al. 2019). The second approach relies on silencing catabolic enzymes or transcription regulators and, accordingly, silencing *C19-GA 2-oxidases*; in tomato, the implementation of this approach induced parthenocarpic development and inhibited lateral branching (Martínez-Bello 2015). The third approach involves spraying plants exogenously with a GA; in GA-treated cherry *Cerasus pseudocerasus*, transcriptome analysis revealed that at anthesis the *DELLA* antagonists, p*avscl1* and *pavscl3*, and the receptor *GID1* were downregulated, whereas *GA2ox* and *DELLA* genes were upregulated after GA treatment, confirming that the exogenous treatment with a GA induced parthenogenesis (Wen et al. 2019).

In the current study—motivated by a desire to develop prickly pear as a crop that can thrive under conditions of global warming and to meet the consumer demand for prickly pear fruits without many hard seeds—we sought to gain an in-depth understanding of GA-based parthenocarpy in prickly pear. We thus studied ovule and seed development in prickly pear both by exploiting the natural genetic parthenocarpic trait of the BS1 variant and by artificial manipulation of GA levels by spraying inhibitors on flower buds. We hypothesized that the BS1 parthenocarpic variant isolated in a stand of *O. ficus-indica* cv ‘Ofer’ plants was generated by spontaneous mutations in genes related to GA biosynthesis/regulation, which led to an imbalance in GA levels. The mutation most likely occurred through transposable elements affecting seed development via modifications in GA regulation and biosynthesis.

## Materials and methods

### Plant material and growth conditions

*O. ficus-indica* BS1 mutant plants were grown on the Bergmann Campus of Ben-Gurion University of the Negev, Beer-Sheva, Israel. Average minimum and maximum temperatures in the summer are 21.6–34.5 °C and 7.7–17.4 °C in the winter. The plants were drip irrigated and fertilized every 3 days in the summer and every 7 days in the winter. The irrigation water contained fertilizer (23N–3P–20 K) at N concentration of 30–40 mg l^−1^ was applied continuously. For the morphological study, a minimum of 20 fruits from mutant and revertant stems were harvested randomly. Fruit size and ovule fresh weight (FW) were determined within 24 h of harvest, and ovule dry weight (DW) was determined following vacuum drying. The effect of 5000 ppm PBZ and 2500 ppm CCC on ovule weight and seed content in BS1 fruit was determined for flower buds, about 1 cm long, sprayed with the GA inhibitors, PBZ, CCC, or control (water containing 0.1% Silwet® L-77) 30 days post bud emergence. Seeds were extracted from 12 fruits. Ovules were sampled from five flowers at 3 days post anthesis. For RNA extraction, flower buds were collected before anthesis (about 5 cm long) and at anthesis. Ovules were lyophilized and stored at −80 °C for RNA extraction and GA analysis.

### Histological studies

Ovules at anthesis were fixed for 24 h in FAA solution (70% ethanol–formalin–glacial acetic acid, 80:10:10, v/v/v) at 4 °C (Cisneros et al. 2011). Dehydration was achieved through a sequence of graded ethanol transfers (60, 70, 80, 90, and 100%), followed by 3 days of infusion at 60 °C in melted Paraplast Plus® (McCormick Scientific). Thereafter, samples were poured into 1-cm^3^ embedding molds, left at room temperature until the Paraplast had thickened, and then transferred to 4 °C for polymerization (Cisneros et al. 2011). A rotating microtome (RM2235, Leica) was used to cut 8-µm sections, which were double-stained with Harris hematoxylin and eosin-Y (Accustain® Harris hematoxylin solution, procedure no. HHS; Sigma–Aldrich). Subsequently, slides were examined with an Axio ImagerA1 microscope with LED illumination (Zeiss) and photographed with an AxioCam HRC camera (Zeiss). Ovules were measured using AxioVision 4.6 (Zeiss).

### GA analysis

Ovules were extracted from flower buds at the pre-anthesis stage when the buds were about 5 cm long or at anthesis (7 cm long). Bioactive GAs, including their inactive biosynthetic precursors, and catabolites were determined in six replicates, following the methodology outlined by Urbanová et al. (2013). The SPE-purified tissue extracts containing internal GA standards purchased from OlChemIm, Czech Republic were analyzed using an ultra-high performance liquid chromatography system (Acquity UPLC™ I-Class PLUS; Waters, Milford, MA, USA) coupled to a triple-stage quadrupole mass spectrometer (Xevo® TQ XS; Micromass, Manchester, UK) equipped with an electrospray interface (ESI). GAs were quantified by the standard isotope dilution method (Rittenberg and Foster 1940). The LC–MS data were acquired and processed using MassLynx™ software (version 4.2, Waters, Milford, MA, USA).

### RNA extraction and cDNA synthesis

Total RNA was extracted following a modified protocol using the Quick-RNA™ Miniprep Kit (Zymo Research, CA, USA). DNase I from the Zymo kit was employed to remove genomic DNA. Subsequently, the purified RNA samples were reverse transcribed into cDNA using the iScript cDNA synthesis kit (BioRad Inc., USA).

### Validation of gene expression

The genes *GA20ox*, *GA13ox*, *GA3ox*, and *GA2ox*, were analyzed using cDNA samples from the pre-anthesis and anthesis stages. Actin was used as the internal reference gene. Primers were designed using Primer-BLAST (NCBI).

### qRT–PCR analyses

qRT–PCR was conducted with ABI 7500 real-time PCR System (Applied Biosystems, CA, USA) using the SYBR® Green PCR Master Mix (Applied Biosystems, CA, USA). For the analysis, 10 µL of reaction mixture contained 2 µL of diluted cDNAs (~ 15 ng/μL), 5 µL of SYBR GREEN PCR Master Mix, 0.3 µL of each primer (10 µM) and 2.7 µL of doubly distilled water. Gene-specific primers were designed using Primer3web (version 4.1.0) (Table S1). The PCR reaction conditions were as follows: 50 °C for 2 min; 95 °C for 10 min; 40 cycles at 95 °C for 15 s; 56 °C for 30 s; and 72 °C for 40 s. The melting curve was generated by heating the amplicon from 60 to 95 °C to confirm primer specificity. Each PCR reaction was repeated three times with four biological replicates. Relative fold changes in gene expression were calculated using the comparative 2^−ΔΔCT^ method.

### Transcriptome and de novo transcriptome assembly

RNA-seq was used to comprehensively examine the differences in transcriptome profiles between the BS1 and revertant phenotypes. The study was conducted using the Illumina HiSeq 4000 platform (Illumina, Chicago, IL, USA). De novo transcriptome assembly was performed using SPAdes version 3.10.1 on raw Illumina reads with default parameters and a k-mer value of 55 (Bankevich et al. 2012). Contigs were filtered after assembly to a minimum length of 200 base pairs. Kallisto was used to calculate transcript expression levels (Bray et al. 2016). A sequence search against the SwissProt database using DIAMOND in blastx mode was used to identify genes and open reading frames (ORFs), followed by KEGG orthology annotation (Buchfink et al. 2015; Zaru and Orchard 2023). The exactTest technique with TMM (trimmed mean of the *M*-values) normalization was used in edgeR for the analysis of differential transcript abundance (Robinson et al. 2010; McCarthy et al. 2012), with false discovery rate (FDR) correction according to Benjamini and Hochberg (1995). The transposon sequences underwent annotation and analysis through CENSOR and RepeatMasker (Bao et al. 2015). The identified transposons were compared to other plant transposon sequences using UniProt tools such as BLAST, alignment, peptide search, and ID mapping (Zaru and Orchard 2023). The RNA-Seq data were visualized and analyzed using IDEP (Ge et al. 2018).

### Evaluation of differentially expressed genes related to GAs

Transcriptome data were used to confirm the expression of DEGs linked to GA (specifically *KAO*,* GID1*, *SCL1*, *SCL13*, and *SCL21*) and the transposons Ty3/Gypsy1, and Ty3/Gypsy2 (Neumann et al. 2019; Jaiswal et al. 2022). Evaluations were conducted utilizing samples from both the pre-anthesis and anthesis stages of the BS1 and revertant. Primers were designed using Primer-BLAST (NCBI) and are listed in the Table S1.

### Statistical analyses

Each treatment involved at least six independent biological replicates for measurement of GAs in ovules (*n* = 6), at least four independent biological replicates for expression analysis (*n* = 4), and three independent biological replicates for transcriptome analysis (*n* = 3). Data are presented as means ± SE were analyzed using Student’s *t*-test, followed by Tukey’s HSD for post-hoc tests (*p* < 0.05).

## Results

### The parthenocarpic BS1 mutant spontaneously reverts to its progenitor phenotype with a high frequency

**Fig. 1:**
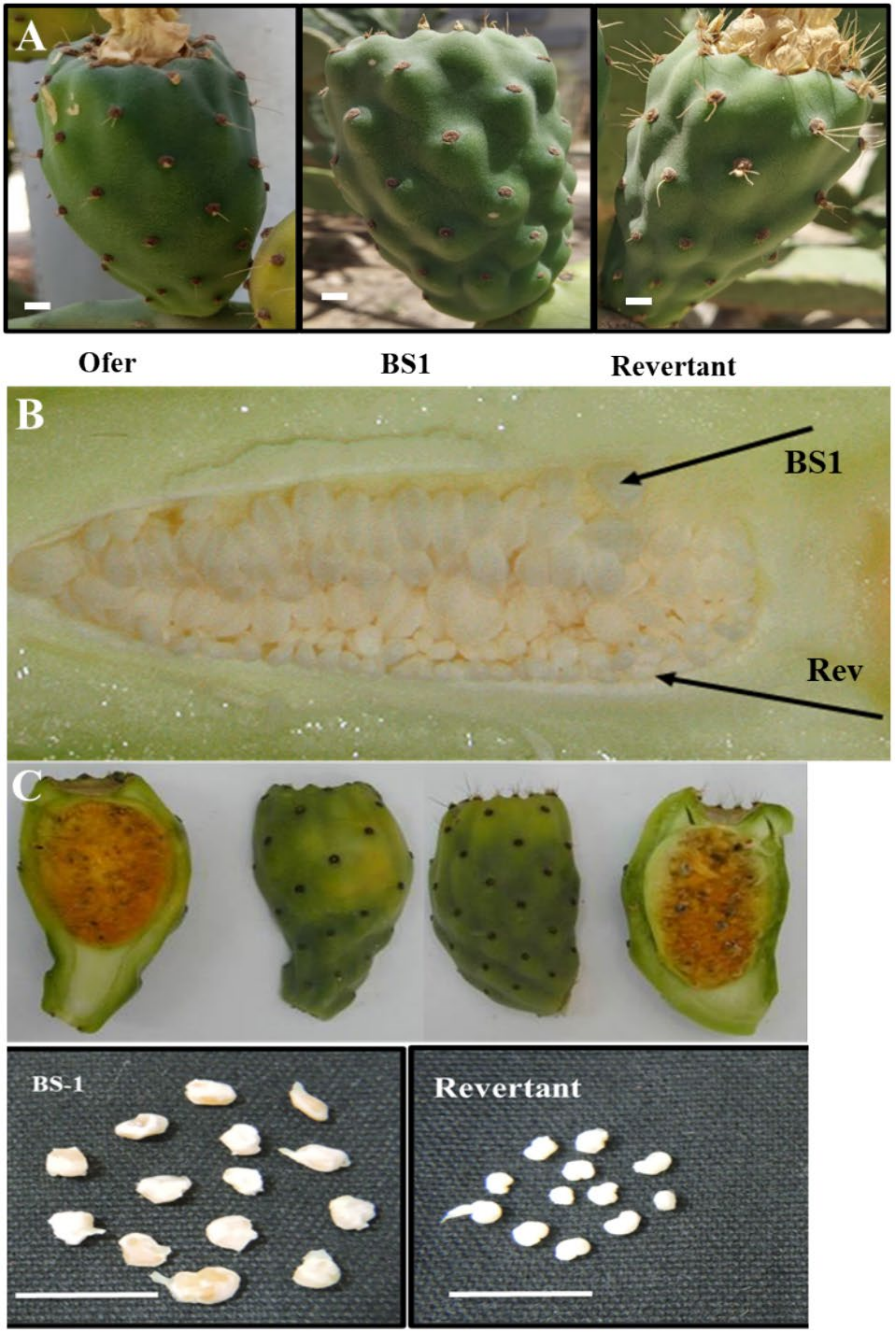
Phenotypes of the mutant BS1, revertant and progenitor cv ‘Ofer’ fruits. **A** Fruit morphology. **B** BS1 flower bud with mixed phenotypes of enlarged ovules and small revertant viable ovules. **C** BS1 fruit (left) with a long neck, large, degenerated seed-like structures, and small viable revertant seeds. Bar 1 cm

BS1 plants produce flowers containing enlarged ovules that develop into small, degenerated seed-like structures and thus bear parthenocarpic (seedless) fruits (Fig. 1A–C). However, in the parthenocarpic fruit numerous unfertilized ovules develop into large hard, lignified brown seed-like structures (Fig. 1C). Among the BS1 plants in our collection, we observed reversion of the mutant phenotype to the progenitor phenotype in many stems (Table 1). The revertant stems were easily identified by the long spines on the flower buds. Some of the BS1 flower buds contained mixed ovule phenotypes arranged in rows; of these, some contained enlarged mutant ovules and others, small normal revertant ovules (Fig. 1B). Mature BS1 fruits had a longer “neck” than the Ofer progenitor fruits and revertant fruits, which have a short normal “neck” (Fig. 1C).

**Table 1:** Frequency of BS1 and revertant phenotypes in stems, flowers and carpels. The frequency of phenotypes was determined in 10 BS1 plants; five stems were sampled from each plant. At least four flowers from each stem were analyzed for ovule phenotyping (giving a total of 250 flowers analyzed). Two stems (together bearing 21 flowers) showed mixed flower phenotypes in the same flower—BS1 phenotype with enlarged ovules, revertant phenotype with small ovules, and dual (BS1/revertant) phenotype. Data for carpels are presented as means ± SE

Plant part	Phenotype	Number	Percentage
Stems	BS1 phenotype stems	43/50	86
	Revertant stems	5/50	10
	Stems with mixed phenotypes	2/50	4
Flowers	BS1 flowers in mixed stems phenotype	9/21	43
	Revertant flowers in mixed stems phenotype	6/21	28
	Mixed flower phenotype in mixed stems phenotype	6/21	28
Carpels	Revertant carpels in mixed flowers phenotype (six flowers)	3.2 ± 0.28	53 ± 4.7
	BS1 carpels in mixed flowers phenotype (six flowers)	2.8 ± 0.28	47 ± 4.7

To determine the reversion rate from the BS1 phenotype to the progenitor phenotype, we screened five stems from each of 10 BS1 plants. Among the 50 screened stems, 43 (86%) bore only the BS1 phenotype fruit (Table 1, Fig. 1C). Of the remaining stems, five stems carried flowers with the revertant phenotype (10%), and two stems (4%) carried flowers with mixed phenotypes inside the flower buds. These flower buds contained rows of BS1 large ovules and rows of revertant normal small ovules. The flower buds containing the mixed ovule phenotypes indicated that a mutation had occurred in the progenitor cells, thus producing rows of ovules with different phenotypes (Fig. 1B). The high rate of spontaneous reversion to the progenitor phenotype implies an active mechanism, possibly involving transposons that alter the levels of plant growth regulators or their regulatory elements during ovule development.

**Fig. 2:**
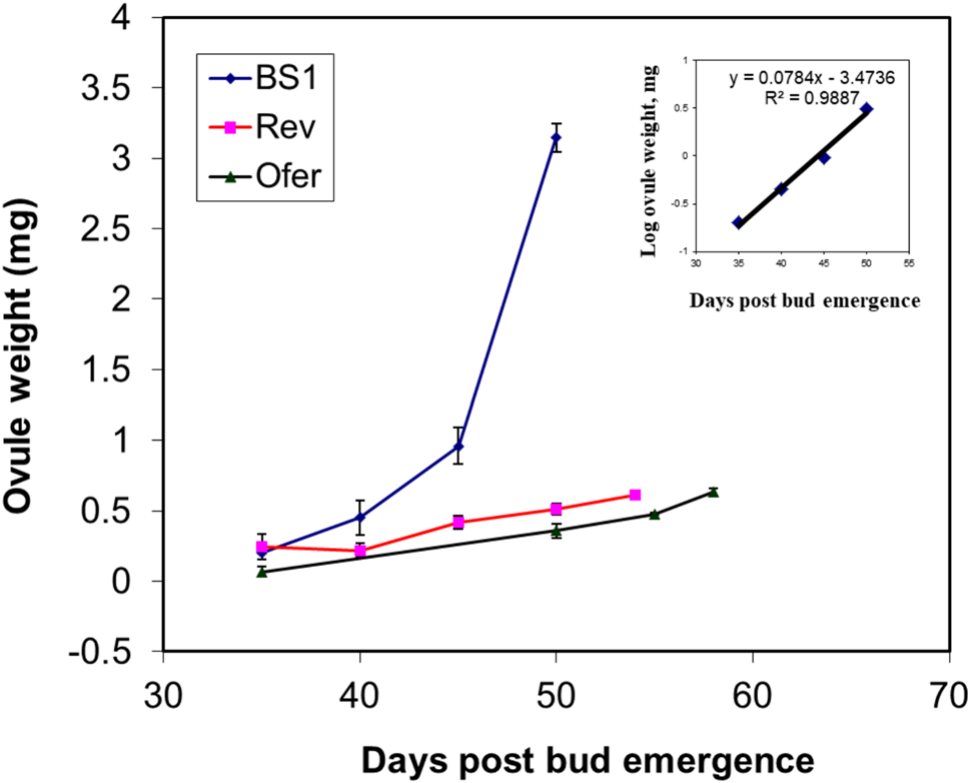
Ovule growth rates in the mutant BS1, revertant, and progenitor cv ‘Ofer’. Ovules were collected over a period starting from the time that they were first visible until anthesis. Ovules were extracted from 3 to 5 flower buds at each sampling date. Data are presented as means ± SE. The inset shows the coefficient of determination for BS1 ovule weight

**Fig. 3:**
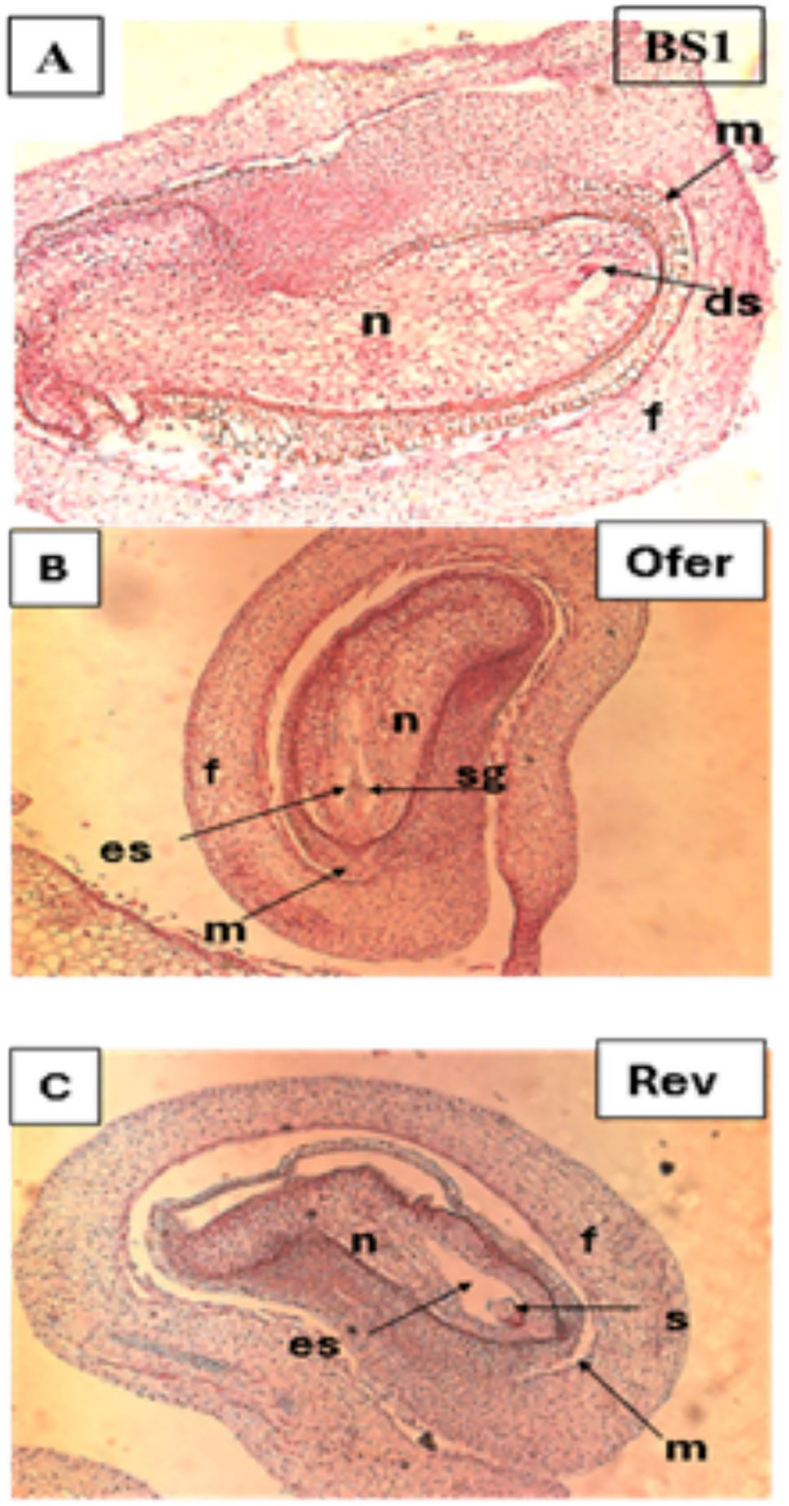
Longitudinal cross sections of ovules of BS1, revertant and the progenitor cv ‘Ofer’. **A** Mature BS1 ovule (1.5 mg) with embryo sac without defined borders. **B** Ofer ovule with embryo sac with clearly defined borders. **C** Revertant ovule with embryo sac with clearly defined borders. ds, degenerated synergids; es, embryo sac; f, funicle; m, micropyle; n, nucellus; s, synergids; sg, starch grains

Examination of the rate of ovule development in BS1 flowers revealed an exponential increase in ovule weight compared to the linear growth rate of ovules in revertant and ‘Ofer’ progenitor plants (Fig. 2). The weight increased mainly between 40 and 50 days post-bud emergence, i.e., about 10 days before anthesis. In addition, BS1 fruits exhibited higher dry ovule weight, fruit weight, and fruit shape index (Figs. S1–S3). Longitudinal cross-sections of ovules revealed that the embryo sac in BS1 ovules did not have well-defined borders. The embryo sac contained degenerated synergid cells and nucellus cells and exhibited thickening of the integument at the micropylar end (Fig. 3A). In contrast, the embryo sacs of the revertant and progenitor cv ‘Ofer’ ovules had clearly defined borders (Fig. 3B and C).

Considering all the data presented above, i.e., the fruit phenotype, large ovule size and early ripening, it is indeed conceivable that an increase in GA content is involved in the specific phenotype of BS1 plants.

### The phenotype of BS1 flowers and ovule degeneration derive from supra-optimal levels of GAs

**Fig. 4:**
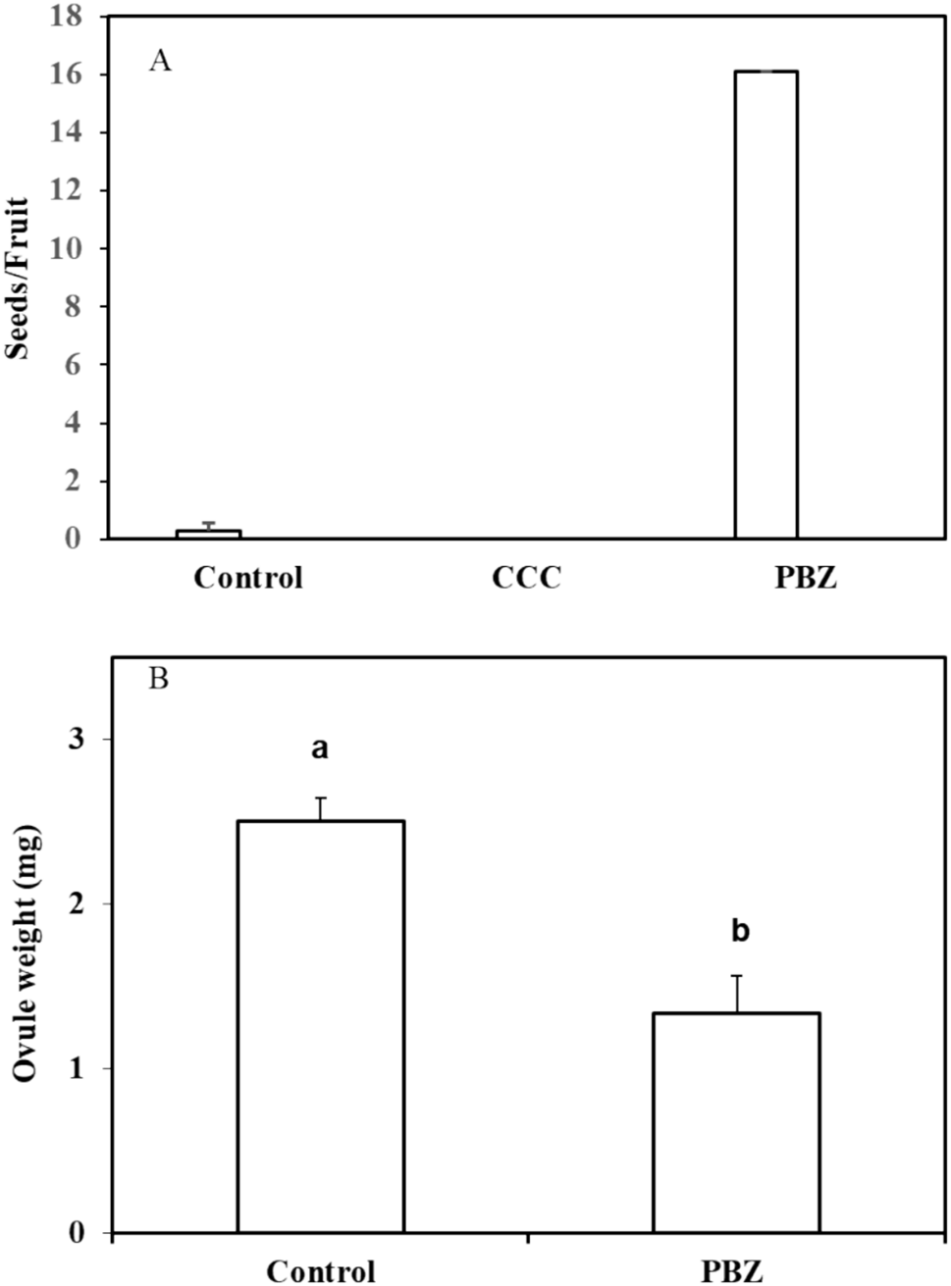
Effect of spraying BS1 flower buds with the GA inhibitors paclobutrazol (PBZ) and chlormequat chloride (CCC), **A** on number of seeds and **B** on ovule weight. Flower buds about 1 cm long were sprayed 30 days post bud emergence with 5000 ppm paclobutrazol, 2500 ppm CCC, or water containing 0.1% of the wetting agent L-77 (control). Seeds were extracted from 12 fruits. Ovules were sampled from five flowers 3 days after anthesis. The data are presented as means ± SE and were analyzed using Student’s *t*-test. Different letters indicate statistically significant difference at *p* < 0.05

Based on our findings for the BS1 phenotype of flowers and fruits presented above, we hypothesized that BS1 fruits contain above-optimal levels of GAs. In keeping with this premise, we found that paclobutrazol (PBZ), an inhibitor blocking the oxidation of *ent*-kaurene, did indeed induce the formation of significant numbers of viable seeds (15/fruit) in BS1 fruits, while control untreated BS1 fruits did not produce any viable seeds (Fig. 4A). A different GA inhibitor, chlormequat chloride (CCC), had no effect on viable seed development, probably due to coticule barrier that prevented efficient penetration of the inhibitor. To further verify the effect on ovule development of reducing GA levels by exogenous PBZ application, the weight of flower buds (pre-anthesis, 1 cm long) that had been sprayed with PBZ was monitored. PBZ significantly reduced the average weight of BS1 ovules to about half that of untreated BS1 control ovules. The average ovule weight determined three days post-anthesis was 2.51 mg vs. 1.34 mg for the untreated control ovules (Fig. 4B). These results thus support the hypothesis that high levels of GAs are responsible for the BS1 phenotype. In light of this validation, endogenous levels of GAs were then determined in both BS1 and revertant ovules.

During pre-anthesis and anthesis, the levels of GA_53_, GA_44_, and GA_19_, which are derived from the early-13-hydroxylation pathway, were significantly higher in the revertant ovules than those in BS1 ovules (Table 2). In particular, the level of GA_53_, the first substrate of the early-13-hydroxylation pathway, was 112-fold higher in revertant ovules than in BS1 ovules during pre-anthesis and 51-fold higher during anthesis. In contrast, the levels of the bioactive GAs, GA_1_ and GA_4_, were significantly higher in BS1 ovules than those in revertant ovules (Table 2). There was no significant difference between BS1 and revertant ovules in the endogenous levels of GA_29_, GA_6_, GA_3_, GA_24_, and GA_34_. These results suggest that higher concentrations of bioactive GAs are available in BS1 and that there are differences between BS1 and revertant plants in the expression of biosynthetic, catabolic, or regulatory genes involved in GA homeostasis.

**Table 2:** Contents of endogenous GAs during ovule development in BS1 and revertant ovules. Ovules were extracted from six flower buds. Data are presented as means ± SE. Mean values in each column followed by the same letters are not significantly different by Student’s *t*-test (*p* < 0.05)

GA metabolite	Content during pre-anthesis phase (pmol/mg DW)		Content during anthesis (pmol/mg DW)	
	Revertant ovules	BS1 ovules	Revertant ovules	BS1 ovules
GA_53_	204.02 ± 20.26^c^	1.82 ± 0.36^b^	98.57 ± 10.37^a^	1.91 ± 0.43^b^
GA_44_	22.94 ± 1.80^c^	6.12 ± 0.99^b^	23.64 ± 0.83^a^	5.15 ± 0.49^b^
GA_19_	66.53 ± 3.33^c^	6.50 ± 1.57^b^	73.93 ± 3.86^a^	5.87 ± 0.83^b^
GA_20_	8.06 ± 1.97^c^	22.82 ± 2.54^b^	7.44 ± 0.76^a^	16.39 ± 0.84^b^
GA_1_	11.67 ± 3.76^c^	103.72 ± 15.51^b^	8.10 ± 1.26^a^	56.45 ± 4.91^b^
GA_4_	0.04 ± 0.05^c^	5.29 ± 0.57^b^	0.44 ± 0.08^a^	6.06 ± 0.24^b^
GA_8_	9.48 ± 0.70^c^	20.58 ± 1.93^b^	9.65 ± 0.51^a^	24.84 ± 0.79^b^
GA_3_	0.11 ± 0.01^b^	0.29 ± 0.01^c^	ND^*^	0.28 ± 0.02^a^
GA_29_	0.91 ± 0.03^b^	0.98 ± 0.05^b^	0.95 ± 0.06^a^	0.84 ± 0.01^a^
GA_9_	0.48 ± 0.09^b^	1.39 ± 0.07^c^	ND^*^	2.19 ± 0.14^a^
GA_5_	ND^*^	ND^*^	ND^*^	ND^*^
GA_6_	0.18 ± 0.01^b^	0.22 ± 0.02^b^	0.22 ± 0.01^a^	0.26 ± 0.03^a^
GA_12_	ND^*^	ND^*^	ND^*^	ND^*^
GA_15_	ND^*^	ND^*^	ND^*^	ND^*^
GA_24_	0.09 ± 0.02^a^	ND^*^	ND^*^	ND^*^
GA_34_	0.03 ± 0.00^c^	0.52 ± 0.06^b^	0.04 ± 0.01^a^	0.69 ± 0.05^b^

^*^ND, not detected; DW, dry weight

Different letters indicate statistically significant difference at* p*0.05

### Differential expression of GA biosynthetic, catabolic, and regulatory genes in BS1 and revertant ovules

To determine the expression levels of GA-related genes, RNA was extracted from BS1 and revertant ovules at pre-anthesis and anthesis. Six libraries were constructed using an Illumina RNA-Seq sequencing platform. A bar plot illustrating total read counts per library was produced (Fig. S4A) and indicated minor differences in library sizes (Supplementary Data Fig. S4B and C). The variation between replicates was also minor (Fig. S4D). A total of 7717 differentially expressed genes (DEGs) were identified; of these 5539 were upregulated, and 2178 were downregulated in BS1 compared to revertant ovules (Figs. S4E–F and S5A–D). KEGG enrichment analysis revealed that DEGs related to the functional category ‘plant hormone signal transduction’ were significantly enriched during flower development (data not shown).

**Fig. 5:**
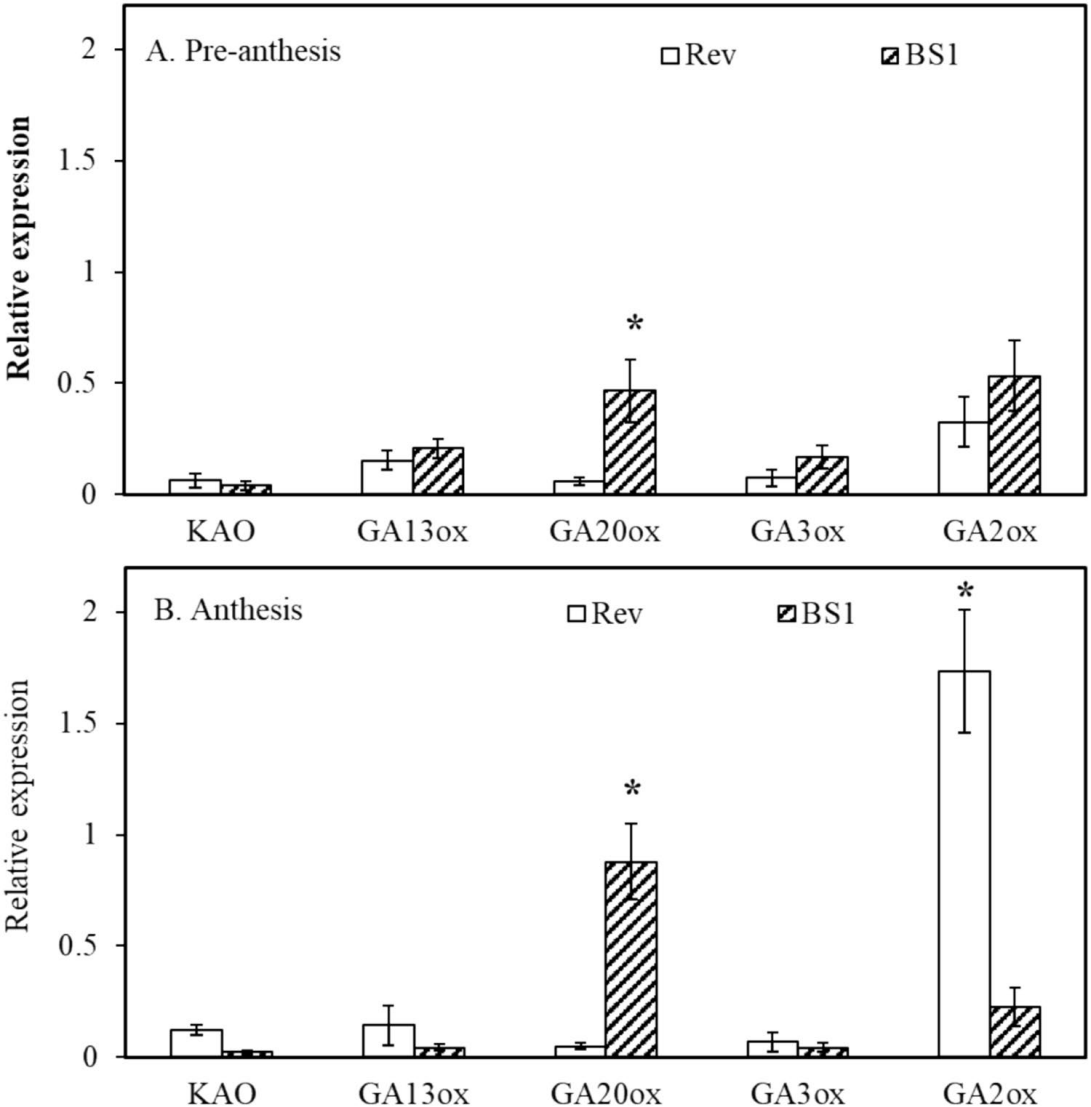
Expression levels of genes coding for enzymes involved in the biosynthetic pathway of GAs in BS1 and revertant (Rev) ovules. Ovules were extracted from bud flowers during **A** pre-anthesis and **B** anthesis. RNA was isolated, and levels of expression were determined by qPCR. Data are presented as means ± SE (*n* = 4) and were analyzed by Student’s *t*-test. The asterisk denotes a statistically significant difference at *p* < 0.05

The expression of key GA-related genes was verified by qPCR; these genes included GA biosynthetic genes (*KAO*, *GA13ox*, *GA20ox*, and *GA3ox*), signaling genes (*GID1*, *SCL1*, *SCL13*, and *SCL21*), and putative transposable elements (*Ty3/Gypsy1* and *Ty3/Gypsy2*). The expression levels of the GA biosynthetic genes, *KAO*, *GA13ox*, and *GA3ox*, were similar at both pre-anthesis and anthesis in BS1 and revertant ovules (Fig. 5A and B). In contrast, expression of the GA biosynthetic gene *GA20ox* was higher in BS1 compared to revertant ovules during both stages of flower development (Fig. 5A and B). Additionally, the expression of the catabolic gene *GA2ox* was significantly higher in revertant ovules at anthesis, which may explain the lower levels of bioactive GAs in revertant ovules.

**Fig. 6:**
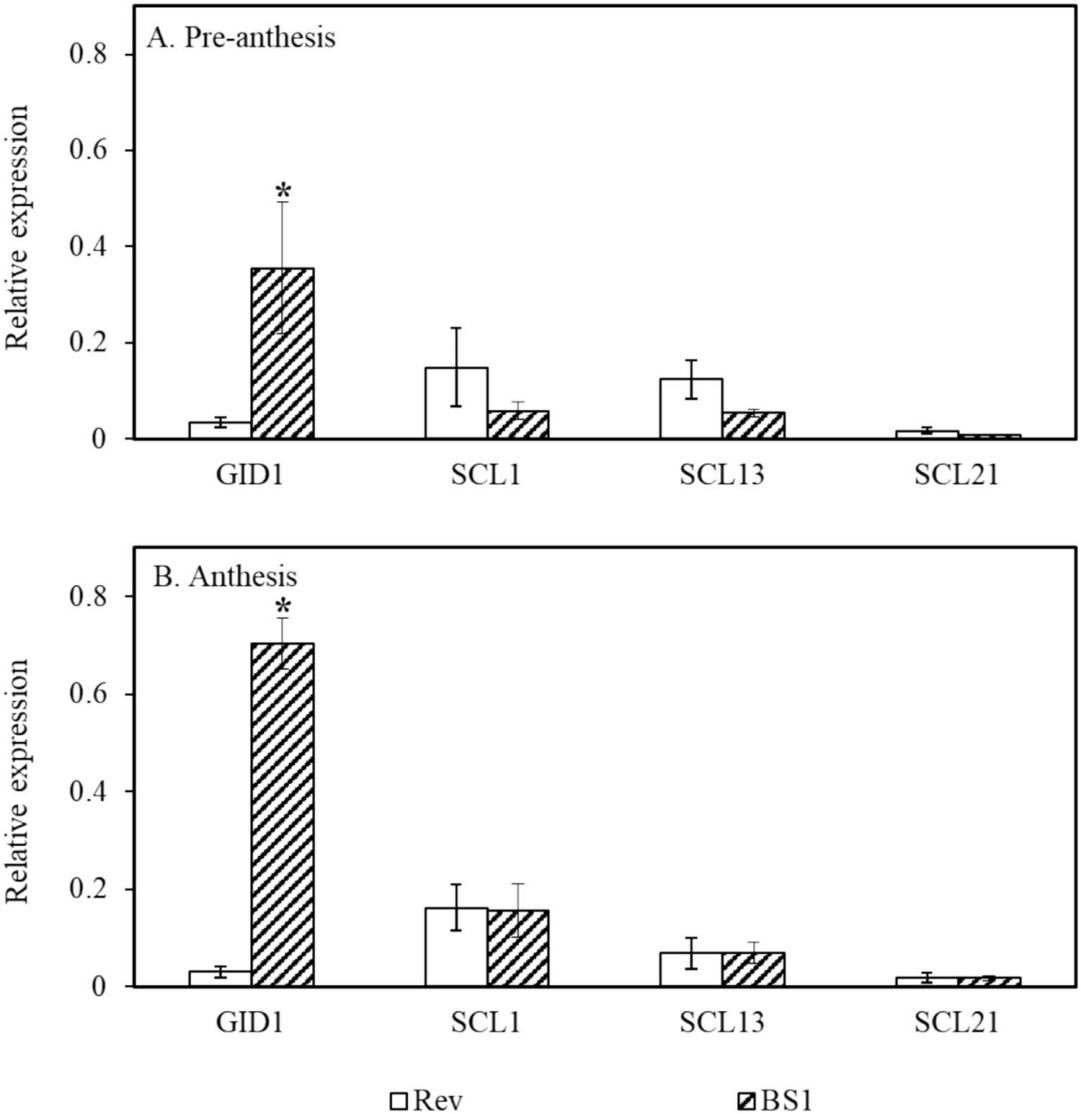
Expression of *GID1* and *SCL* genes involved in signaling and regulation of GAs in BS1 and revertant (Rev) ovules during flower bud development. Ovules were extracted from BS1 and revertant flower buds during pre-anthesis and anthesis. RNA was isolated, and levels of expression were determined by qPCR. Data are presented as means ± SE (*n* = 4) and were analyzed using Student’s *t*-test. The asterisk denotes a statistically significant difference at *p* < 0.05

Analyses of the regulatory genes revealed that expression of *GID1*, the GA receptor, was significantly higher in BS1 ovules than in revertant ovules at both flowering stages (Fig. 6A and B). The expression of the transcriptional regulators of the family of GAI-RGA-SCR (GRAS) proteins was also determined, and the sequences of the prickly pear genes were named based on homology to *SCL* genes. The expression of *SCL* genes was higher—but not statistically significantly higher—in revertant ovules at the pre-anthesis stage (Fig. 6A and B). During anthesis the expression of putative *SCL* genes was similar in BS1 and revertant flowers.

**Fig. 7:**
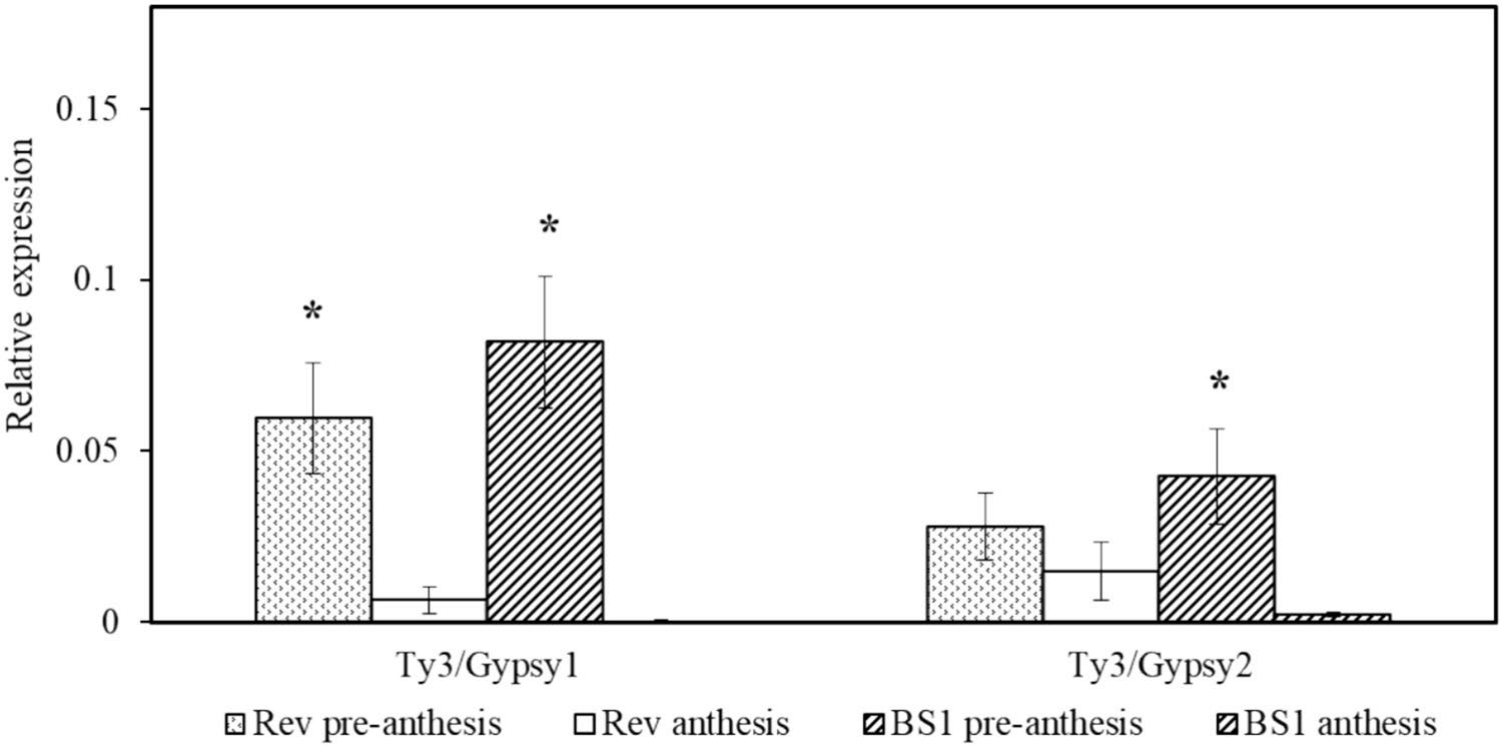
Expression levels of two putative transposons in BS1 and revertant (Rev) ovules during flower development. RNA was isolated from ovules during pre-anthesis and anthesis, and levels of expression were determined by qPCR. Data are presented as means ± SE (*n* = 4) and were analyzed using Student’s *t*-test. The asterisk denotes a statistically significant difference at *p* < 0.05

The high frequency of the revertant phenotype (10%, Table 1) in the population of BS1 flowers led us to assume that active transposons are involved in the formation of the BS1 phenotype. Indeed, a search of the transcriptome data revealed two putative long terminal repeat (LTR)–retrotransposon sequences expressed in BS1 and revertant ovules (Fig. S6A–C). The sequences shared ~ 70% homology with the Gypsy-type family. Notably, the expression of the retrotransposons in both BS1 and revertant ovules was significantly higher during the pre-anthesis stage of the flower development (Fig. 7). However, the expression of the putative transposons was lower at anthesis than during pre-anthesis. If the transposon activity affects the expression of genes involved in GA regulation and parthenocarpic fruit development it is expected that higher activity would be at the pre-anthesis stage.

## Discussion

### High levels of GAs affect the phenology of BS1 flowers

Fruit development is triggered by the fertilization of the ovule, which involves a coordinated process of seed development and maturation (Dorcey et al. 2009; Wang et al. 2020). GAs appear to regulate many aspects of flower, ovary and ovule development and seed quantity, as demonstrated in *Arabidopsis* (Gomez et al. 2018, 2020). GAs play roles in stimulating early flowering, reducing ovule numbers, and facilitating the formation of seedless fruits (Gomez et al. 2018, 2020). In the prickly pear mutant, BS1, high levels of GAs affect the phenology and morphology of the fruit (Fig. 1 and S2). Seeds, as a source of hormones, play a pivotal role in the typical development of fruits, influencing their size and shape as a function of seed number and distribution. *O. ficus-indica* fruits with viable seeds have an oval shape, while those with aborted seeds often appear elongated due to pulp tissue regeneration (Abo-El-Ez et al. 2023). Accordingly, BS1 mutant fruits are larger and exhibit a longer neck than revertant fruits (Fig. 1), and BS1 flowers are much larger than the revertant flowers (Fig. S3). BS1 parthenocarpic fruits, compared to revertant fruits, are also characterized by early ripening, larger ovules, and fewer, smaller spines (Figs. S1 and S2). The distinctive fruit shape and the occurrence of parthenocarpy in BS1 vs. revertant fruit may be attributed mainly to elevated levels of certain GAs (Table 2). It is indeed known that GAs influence plant morphology; for example, in parthenocarpic apples they are responsible for increases in fruit size, and in parthenocarpic tomatoes, for enlarged ovaries, embryo sacs and larger fruits (Fos et al. 2000; Galimba et al. 2019; Chen et al. 2020). Like BS1 ovules, GA_3_-treated tomato ovules showed limited growth in some cases, but most of them degenerated (Serrani et al. 2007). In BS1 flowers, the ovules are enlarged as an outcome of integument proliferation. The over-growth of the integument probably blocks the micropyle end, thereby preventing the pollen tube from penetrating into the embryo sac and hence precluding fertilization. Similar enlarged ovule phenotypes were also observed in parthenocarpic *Arabidopsis* and tomato due to the proliferation of ovule integument cells (Kasahara et al. 2016; Gupta et al. 2021). It should be noted that when PBZ, the inhibitor blocking the oxidation of *ent*-kaurene, was applied to BS1 flowers, to reduce the endogenous GA levels, viable seeds were obtained, and they germinated properly.

Among the GA-induced morphological traits of BS1 is the activation of primordia that develop into spines; in BS1, these spines are morphologically distinct, since they are smaller (Fig. 1) and less numerous than the spines in the wild type (Mauseth 1977; Corrales-García and González-Martínez 2001). However, when BS1 reverts, it resembles the ‘Ofer’ fruit phenotype in terms of the spines (in numbers and length) (Fig. 1). Overall, the observed morphological differences of the BS1 fruit (vs. revertant and ‘Ofer’ fruit) can be explained by higher concentrations of active GAs in BS1 ovules (Table 2).

### Products of the GA early 13-hydroxylation pathway are ubiquitous in prickly pear ovules

GA-induced parthenocarpic fruit development has been described in a variety of plant species, including *O. amyclaea* (Mejía and Cantwell 2003), *Malus domestica* (Galimba et al. 2019), *P. bretschneideri* (Liu et al. 2018; Wang et al. 2020), and *S. lycopersicum* (Mignolli et al. 2015). In grapes—a classic example—GA treatment or natural mutations lead to elevated GA levels during berry development and to stenospermocarpy (Cheng et al. 2013). In various fruits, different GA biosynthetic pathways are active during development, and several GA derivatives induce parthenocarpy. In higher plants, GA_1_, GA_3_, GA_4_, and GA_7_ are the most prevalent active forms that promote plant growth and development (Wang et al. 2020). In plants such as pea, lettuce, rice, and spinach*,* GA_1_ is the predominant GA, whereas GA_4_ is the predominant active form in species like cucumber and *Arabidopsis* (Eriksson et al. 2006; Pimenta and Lange 2016). In prickly pear, the early 13-hydroxylated GAs are more prevalent than the non-13-hydroxylated GAs (Table 2), similarly to tomato, in which active GAs are generated primarily through the early 13-hydroxylated pathway (García-Hurtado et al. 2012). Although the early 13-hydroxylation pathway is active in BS1 during anthesis, GA_3_ was not detected in revertant ovules, although BS1 ovules did contain some GA_3_ (Table 2). In contrast, in *Arabidopsis*, the principal biologically active GA_4_ is produced through the non-13-hydroxylation pathway (Varbanova et al. 2007; Magome et al. 2013). In prickly pear, *Arabidopsis* and rice GA_1_ and GA_4_, as bioactive GA forms, can be synthesized in both branches of the terpenoid pathway, depending on the tissue and the GA function. For instance, GA_1_, produced by the early-13-hydroxylation pathway, is dominant in *Arabidopsis* siliques, but GA_4_, which is formed through the non-13-hydroxylation pathway, is prevalent in most tissues of *Arabidopsis* (Varbanova et al. 2007; Magome et al. 2013). In contrast, in rice, GA_1_ is the most physiologically active form detected in vegetative tissues, but significantly elevated levels of GA_4_ are found in anthers (Hirano et al. 2008; Magome et al. 2013). In seedless citrus fruits, high levels of GA_1_ and GA_4_ have been linked to early fruit development and the parthenocarpic characteristic (Talón et al. 1990). In grapefruit, as in BS1, GA_1_ is the primary bioactive GA during flower opening, while GA_4_ becomes prevalent at later stages of fruit development (Giacomelli et al. 2013). In *Arabidopsis,* at the time of flowering, the levels of GA_4_ were found to be higher than those of any of the other bioactive GAs regulating flower initiation and elongation (Eriksson et al. 2006). In prickly pear, the predominant bioactive GAs are GA_1_ and GA_4_ (Table 2) although the level of GA_1_ is 20-fold higher than GA_4._ Nevertheless, GID1 receptor has the highest affinity for GA_4_, resulting in a more active complex than the complex with GA_1_ (Ueguchi-Tanaka et al. 2005; Nakajima et al. 2006). Compared to revertant ovules, GA_1_ and GA_4_ levels are higher in BS1 ovules, and the increase in BS1 fruits size is associated with the increased GA_1_ concentration. This conclusion is supported by our experiments that showed that the phenotype of BS1 flower buds treated with the GA inhibitor PBZ reverted to the phenotype of the seeded progenitor ‘Ofer’ (Fig. 4). It is also indirectly supported by a study reporting that a drop in GA_1_ led to reduced fruit growth in tomatoes (Serrani et al. 2007). Similar outcomes were observed in tomatoes treated with PBZ (Chen et al. 2020), where tomato fruit size, shape and elongation depended on reduction of GA concentration by PBZ. These findings further corroborate our experimental results, indicating the significant role of GAs in ovule development and in inducing parthenocarpic fruit in BS1. Taken together, our findings indicate that elevated concentrations of bioactive GAs in ovules are the primary factor governing the development of parthenocarpic fruit in prickly pear plants.

### Overexpression of GID1 and GA20ox and suppression of GA2ox lead to higher levels of GAs

As mentioned above, the levels of biologically active GAs are controlled by the expression of genes involved in GA synthesis and catabolism, but GA levels in tissue are also influenced by the GA signaling pathway (Hedden 2020). High endogenous contents of active GAs in BS1 ovules (Table 2) may be attributed to the high expression of GA20ox and of GID1, which also functions as an activator of GA biosynthesis genes, such as GA20ox and GA3ox (Hedden 2020), combined with the simultaneous suppression of GA2ox expression. Our findings revealed higher GID1 expression in BS1 ovules (vs. revertant ovules), which activates the synthesis of the bioactive GAs, GA_1_ and GA_4_ (Table 2). The higher level of GID1 expression and GAs content found in BS1 ovules support our hypothesis that they are directly involved in parthenocarpic fruit development, as in *Arabidopsis*, in which GID1 expression is crucial for the formation and development of the fruit (Gallego‐Giraldo et al. 2014).

In parthenocarpic tomato and *Arabidopsis,* fruit growth is promoted by a reduction in DELLA repressor activity (Martí et al. 2007; Fuentes et al. 2012). Previous studies in *A. thaliana* seedlings recognized the SCL3 transcription factor as a direct target gene for repression by DELLA (Zentella et al. 2007; Zhang et al. 2011). SCL3 activates GA signaling and accumulation, as demonstrated in *scl3* mutants of *Arabidopsis* in which GA biosynthetic genes were downregulated and GA catabolic genes were upregulated (Zentella et al. 2007; Zhang et al. 2011). In BS1 ovules, vs. revertant ovules, the expression of *SCL1*-like, *SCL13*-like, and *SCL21*-like genes was higher—but not statistically significantly—during pre-anthesis (Fig. 6).

The data presented here (Fig. 6) indicate that in BS1 ovules the upregulated *GID1* expression probably elevates the expression of *GA20ox* genes (Fig. 5). These findings imply that induction of parthenocarpy in BS1 is the outcome of a combination of upregulation of *GID1* receptor expression and upregulation of *GA20ox* expression, with simultaneous repression of the GA catabolic gene *GA2ox* (Figs. 5 and 6). In contrast, the seeded revertant phenotype exhibited an increase in *GA2ox* expression and a decrease in *GID1* and *GA20ox* expression (Figs. 5 and 6).

The instability of the BS1 phenotype and the frequent reversion to normal ovules, and the development of viable seeds imply that transposon activity could be the active mechanism involved in the modification of GA signaling or biosynthesis in BS1 (Table 1, Fig. 7). Transposons are known to regulate gene expression in various plant species (Feschotte et al. 2002; Tatsuki et al. 2018; Niu et al. 2019). In our study, we found sequences that show homology to putative Ty3/Gypsy 1-like and Ty3/Gypsy 2-like transposons, which may play a role in GA homeostasis in BS1 ovules. Sequence analysis revealed significant similarities (Fig. S6) between the prickly pear putative transposons (Ty3/Gypsy 1 and Ty3/Gypsy 2) and transposons from other plant species, such as in parthenocarpic apple (dem1), pineapple (dea1), rice (rire3), and lily (del1) (Yao et al. 2001). The apple transposon dem1 has been shown to regulate parthenocarpic fruit production by modulating the MADS-box transcription factor (Yao et al. 2001; Tatsuki et al. 2018). We propose that active transposons influence parthenocarpic fruit induction by modulating GA biosynthesis or regulation. This notion is supported by the high rate of reversion to the progenitor phenotype in BS1 plants—a rate that is far higher than that of natural spontaneous mutation (Table 1). During evolution transposon played an important role in genome reorganization, occasionally with beneficial effects such as in this case by reducing seeds number. However, further research is necessary to elucidate the precise mechanism by which Ty3/Gypsy 1 and 2 putative transposons are involved in GA regulation of ovule development and to identify the insertion/excision site in the BS1 genome.

**Fig. 8:**
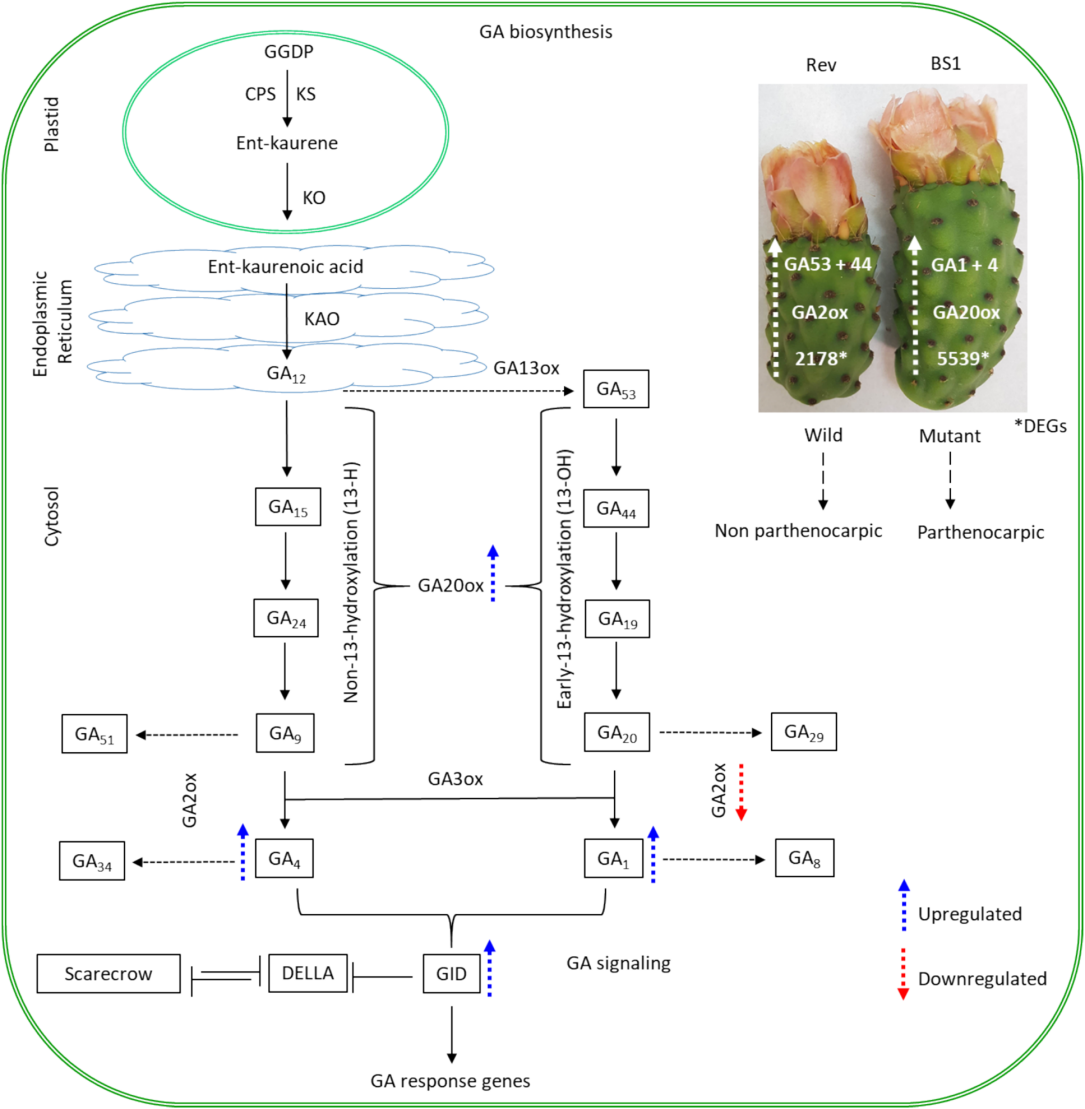
Model proposed for the regulatory network of parthenocarpic fruit development. The excessive growth of unfertilized ovules is induced by elevated contents of GAs due to increased GA20ox expression, leading to accumulation of active GAs, accompanied by suppression of the catabolic enzyme GA2ox and increased expression of the GID1 receptor. Supra-optimal levels of GAs lead to over-growth of the integuments and blocking of the micropillar cavity, which prevents penetration of the pollen tube and, hence, ovule fertilization

In conclusion we propose that the role of GAs in parthenocarpic BS1 fruit development is controlled by the GID-GA20ox/GA2ox genetic system. BS1 ovules contained higher levels of the bioactive GAs, GA_1_ and GA_4_, than revertant ovules due to increased GA2ox and GID1 expression and decreased expression of the catabolic enzyme, GA2ox. Recent work demonstrates that parthenocarpic fruit development in prickly pear requires the involvement of several plant growth regulators including GA, cytokinin and auxin (Livera-Muñoz et al. 2024). Thus, corroborating our findings, that GAs is a pivotal hormone in parthenocarpy development. However, GA is not a single player since profound changes in brassinosteroids content were also detected in parthenocarpic prickly pear fruits (Ramakrishnan et al. 2025). Our study provides valuable insights into the regulatory mechanisms governing parthenocarpic fruit development in prickly pear (Fig. 8). Overall, this study advances the understanding of the hormonal regulation of ovule development in prickly pear and sets the stage for future research aimed at improving fruit quality and consumer appeal—as reflected mainly in the absence of hard seeds—through targeted manipulation of GA signaling pathways. In future when transformation system in prickly pear is available it will pave the way to produce seedless fruits by manipulating the GA biosynthetic pathway.

## Supplementary Information

Below is the link to the electronic supplementary material.

Supplementary file1 (PDF 944 KB)

## Data Availability

The data supporting the findings are accessible both within the paper and in the supplementary materials available online. Data will be shared on request to the corresponding author.
