# Supplementary material for: Gibberellin-induced parthenocarpy in fruits of a prickly pear mutant
Source: Plant Cell Rep. 2025 Aug 6;44(9):194. doi: 10.1007/s00299-025-03568-w (PMC12328502; doi:10.1007/s00299-025-03568-w)
Supplement: Supplementary file 1 — Supplementary file1 (PDF 944 KB) [file 299_2025_3568_MOESM1_ESM.pdf]

Gibberellin-induced parthenocarpy in fruits of a prickly pear mutant

Rameshkumar Ramakrishnan[1], Udi Zurgil[1], Shamili Kanna[1], Danuše Tarkowská[2], Ondřej Novák[2], Miroslav Strnad[2], Noemi Tel-Zur[1]* and Yaron Sitrit[1,3]*

Table S1. List of primers used in this study.

No.	Gene name	Primer (Forward/Reverse)
1	KAO	CCTGGTGATCTGGGTTTGCC<br>CCGTCTTGCCAAACCTGGTG
2	GA13ox	CAAAAGGGTTCGCTCCAAGC<br>CCAGTTGGGTCATCCGAAGG
3	GA3ox	AAATCCCACACTATGCCCGC<br>CCCATTTGAACGAGTCGGGT
4	GA20ox	GGCCGGATGTGTTCCGATTA<br>CGTCGACCCCTCTTCAATCC
5	GA2ox	GGGGCAGTATCAGAGTTGGC<br>GGAGGATGGAGTCGCTTTGG
6	GID	GACGTGCCCCTGAAAACAGA<br>CCACCCGAACTATCACCTGC
7	SCL1	TGGGGTAAGCCCATCTCCTG<br>CGGGCCCTATAACCACCTCA
8	SCL13	CCCTTTGGGAAATGGAGGGC<br>GATAATCCTGCACCCGCACC
9	SCL21	ATGGCAGGGTTCCGACAGTA<br>TGAAGCCGAAACCAGCATCC
10	Ty3/Gypsy1	TTTCAAGGAGGTGCCGATCC<br>GTCCCCCACCAGTATTCAGC
11	Ty3/Gypsy2	CTTGTATCCCAGAAGCGCGA<br>AGGGCCAGCTTCCTAGATCA

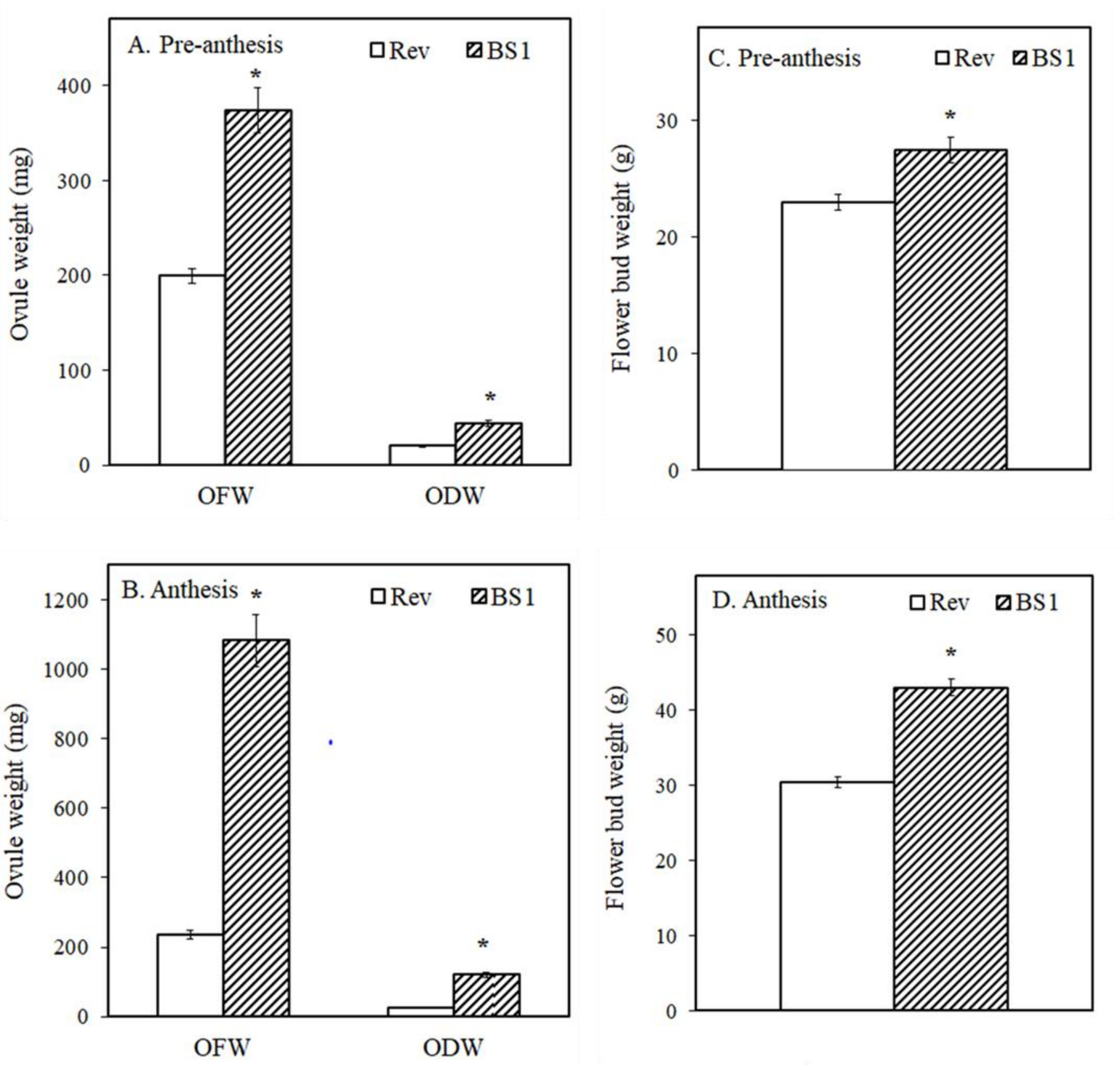

Figure S1. Ovule fresh weight (OFW), ovule dry weight (ODW) and flower bud weight in BS1 and revertant flowers. Ovules were harvested from flower buds at pre-anthesis (A&C) and during anthesis (B&D). Data are presented as means ± SE (n=10) and were analyzed using Student's t-test. Asterisks denote a statistically significant difference at $p<0.05$.

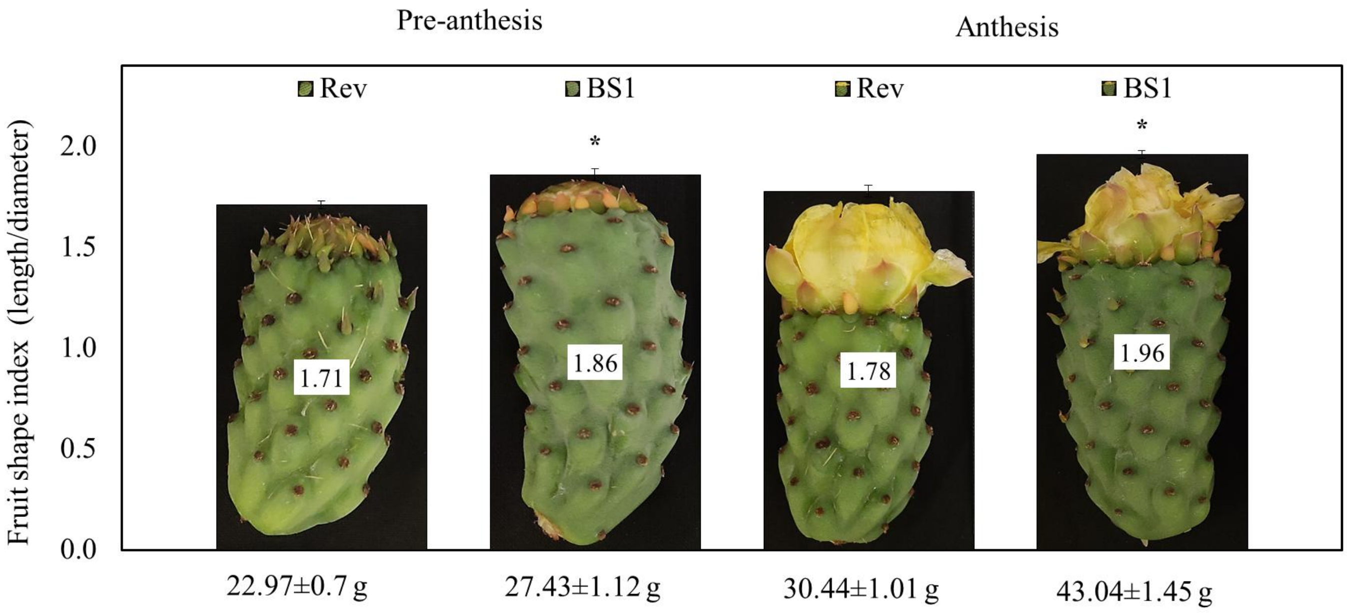

Figure S2. Shape index and weights of BS1 and revertant flower buds during pre-anthesis and anthesis. Numbers on the fruit denote value of the shape index. Data are presented as means ± SE (n=10) and were analyzed using Student's t-test. The asterisks denote statistically significant difference at $p<0.05$.

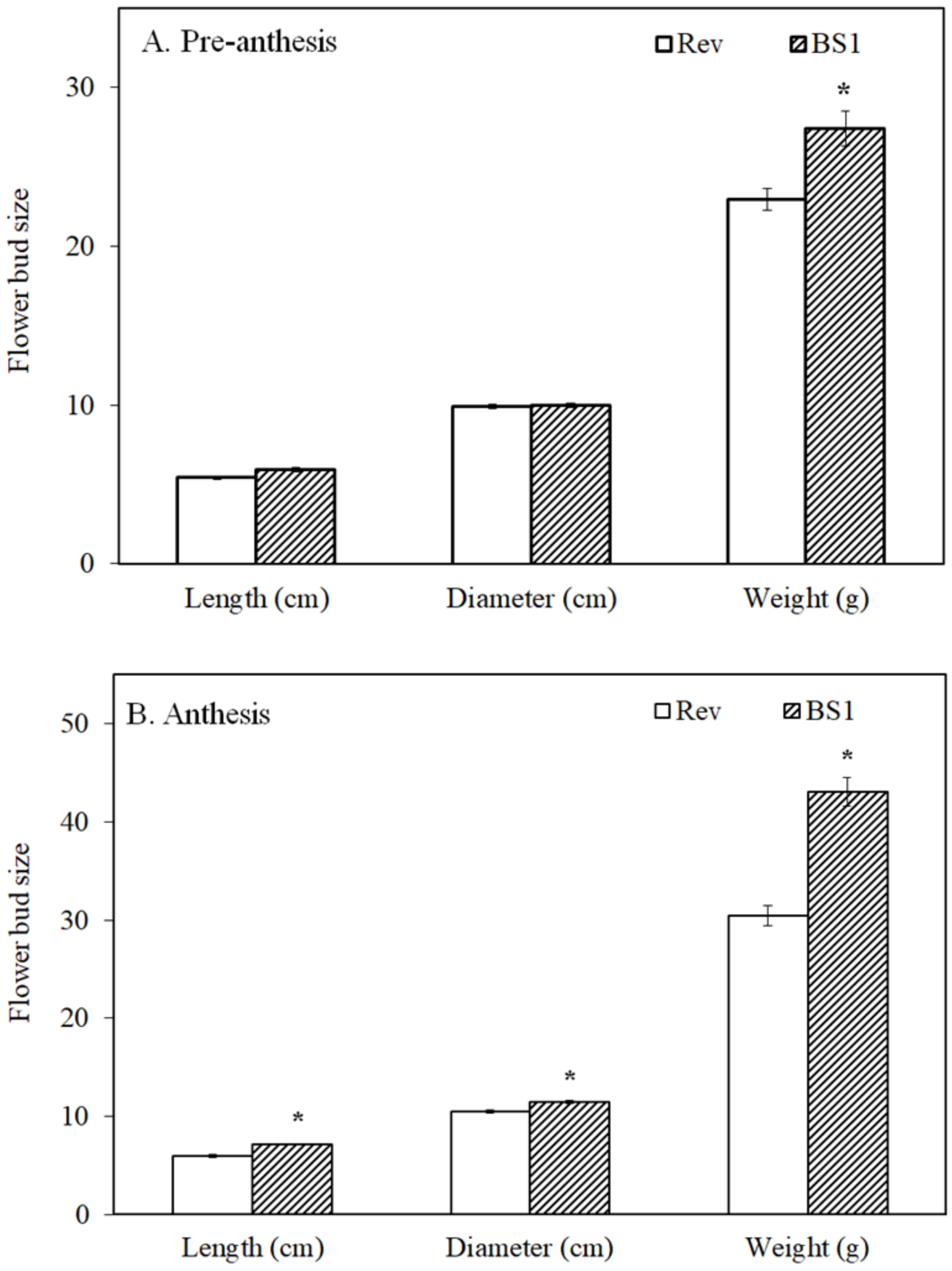

Figure S3. Length, diameter, and weight of BS1 and revertant flower buds during pre-anthesis (A) and anthesis (B). Data for 10 fruits are presented as means ± SE (n=10) and were analyzed using Student's t-test. The asterisks denote statistically significant difference at $p<0.05$.

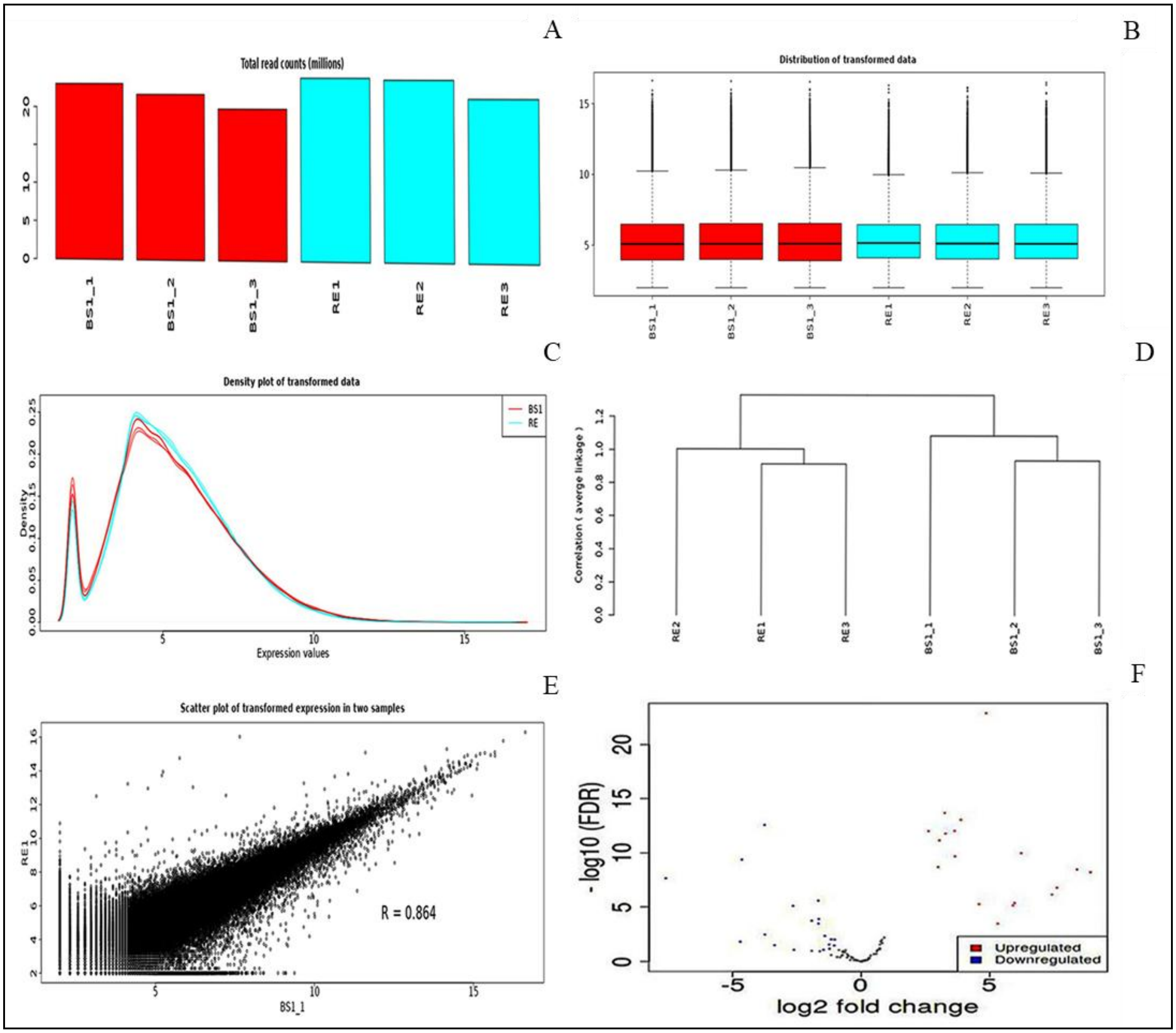

Figure S4. Transcriptome analysis: **A** Bar plot for read counts data showing the variations in read counts between the BS1 and revertant (Rev) groups; **B** Box plot showing a comparative analysis of data dispersion within and between the BS1 and revertant groups for transformed data distribution; **C** Density plot of transformed data showing the differences in data distribution between the BS1 and Rev groups; **D** Phylogenetic tree showing the genetic relationships between BS1 and Rev groups; **E** Scatter plot of transformed expression in BS1 and Rev, showing a strong positive relationship; **F** Volcano plot of differentially expressed genes.

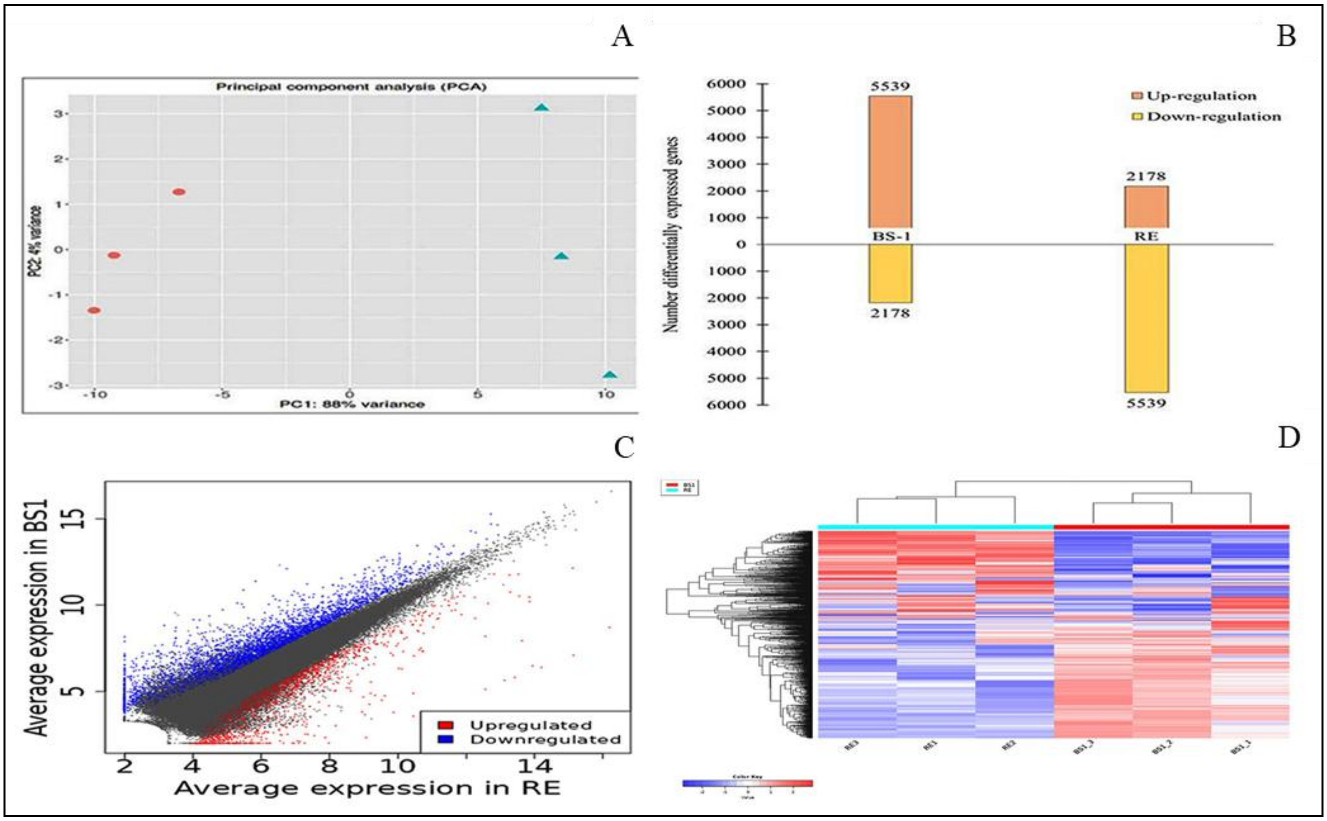

Figure S5. Differential expression of genes in BS1 and revertant (Rev). **A** PCA analysis showing the significant pattern of variation between BS1 and Rev; **B** Total number of DEGs between BS1 and Rev; **C** Mean-Absolute (MA) plot showing DEGs. Log-fold change between BS1 against the mean (M) expression across all Rev samples (A); **D** Hierarchical clustering of DEGs shows the gene expression pattern and relationships between BS1 and Rev samples.

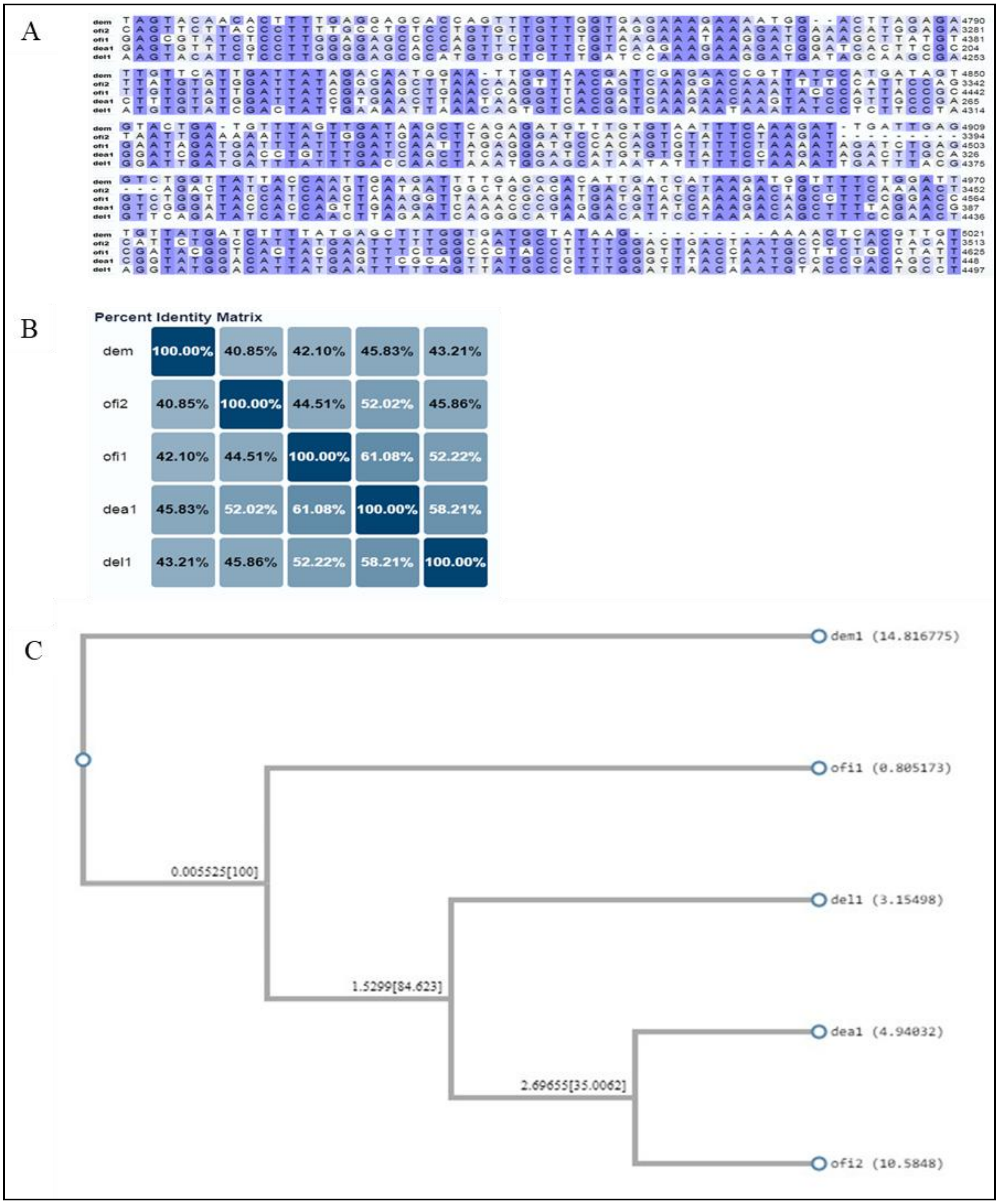

Figure S6. Sequence homology analysis for putative BS1 transposons [ofi1 and ofi2; *Opuntia ficus-indica* (ofi)] and Ty3/Gypsy plant transposons. **A** Sequence homology analysis reveals significant similarities between putative BS1 transposons and transposons from other plant species, including apple (dem1), pineapple (dea1), and lily (del1). **B** Percent identity matrix showing a high percentage of similarities between the transposons. **C** Phylogenetic relationship of the transposons.
